# Lactic Acid Bacteria–Gut-Microbiota-Mediated Intervention towards Inflammatory Bowel Disease

**DOI:** 10.3390/microorganisms12091864

**Published:** 2024-09-09

**Authors:** Diantong Li, Zhenjiang Liu, Xueni Fan, Tingting Zhao, Dongxu Wen, Xiaodan Huang, Bin Li

**Affiliations:** 1 Institute of Animal Husbandry and Veterinary, Xizang Academy of Agricultural and Animal Husbandry Sciences, Key Laboratory of Animal Genetics and Breeding on Tibetan Plateau, Ministry of Agriculture and Rural Affairs, Lhasa 850000, China; 220220912461@lzu.edu.cn (D.L.); zhenjliu@jlu.edu.cn (Z.L.); fanxn21@lzu.edu.cn (X.F.); 220220913170@lzu.edu.cn (T.Z.); xznmwdx@163.com (D.W.); 2 School of Public Health, Lanzhou University, Lanzhou 730000, China; 3 National Engineering Laboratory for AIDS Vaccine, School of Life Sciences, Jilin University, Changchun 130012, China

**Keywords:** inflammatory bowel disease, ulcerative colitis, Crohn’s disease, lactic acid bacteria

## Abstract

Inflammatory bowel disease (IBD), encompassing ulcerative colitis (UC) and Crohn’s disease (CD), arises from intricate interactions involving genetics, environment, and pharmaceuticals with an ambiguous pathogenic mechanism. Recently, there has been an increasing utilization of lactic acid bacteria (LAB) in managing IBD, attributed to their ability to enhance intestinal barrier function, mitigate inflammatory responses, and modulate gut microbiota. This review initiates by elucidating the pathogenesis of IBD and its determinants, followed by an exploration of the mechanisms underlying LAB therapy in UC and CD. Special attention is directed towards their influence on intestinal barrier function and homeostasis regulated by gut microbiota. Furthermore, the review investigates the complex interplay among pivotal gut microbiota, metabolites, and pathways associated with inflammation. Moreover, it underscores the limitations of LAB in treating IBD, particularly in light of their varying roles in UC and CD. This comprehensive analysis endeavors to offer insights for the optimized application of LAB in IBD therapy.

## 1. Introduction

**Figure 1:**
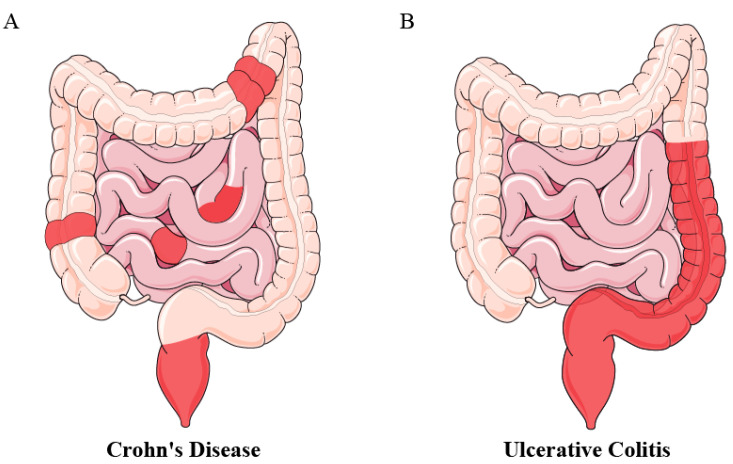
Types of Inflammatory Bowel Disease. The red areas indicate inflammation, with CD (**A**) showing segmental distribution and UC (**B**) presenting as a continuous lesion.

Inflammatory bowel disease (IBD), encompassing Crohn’s disease (CD) and ulcerative colitis (UC), is characterized by persistent inflammation within the small intestine and colon. This condition is multifactorial, with genetic, environmental, and host-related factors contributing to its pathogenesis [1,2]. Though both are classified as IBD, UC and CD differ significantly in terms of regional involvement and the depth of tissue inflammation [3]. UC primarily affects the colon and rectum, with inflammation typically restricted to the mucosal layer. The disease exhibits a continuous pattern of inflammation that starts in the rectum and extends proximally [4]. In contrast, CD can affect any part of the gastrointestinal tract, from the mouth to the anus, and is characterized by a discontinuous or “skip lesion” pattern. CD features transmural inflammation, which can lead to complications such as strictures, fistulas, and granuloma formation (Figure 1) [3].

The clinical heterogeneity of UC and CD complicates the identification of a universally optimal therapeutic strategy, necessitating personalized treatment approaches to cater to the varied presentations across patients [5]. The current therapeutic landscape for IBD is diverse, comprising aminosalicylates, corticosteroids, immunomodulators, antibiotics, biologic agents, Janus kinase inhibitors, and dietary modifications [6]. Wehkamp et al. [7] have underscored the significance of mucosal barrier disruptions, induced by dysbiosis of the bowel microflora, in the etiology of IBD [8,9]. This recognition has spurred the development of novel preventive strategies aimed at rectifying microbial imbalances through conventional therapeutics [10], biologics [11], and novel small molecule drugs [12].

In addition, increasing evidence indicates that probiotics possess therapeutic and prophylactic potential in managing gastrointestinal disorders. These beneficial effects are linked to their ability to modulate the host’s gut microbiota, enhance intestinal barrier integrity, strengthen the immune defense, and regulate inflammatory cytokine levels [7,13,14,15]. Among probiotics, lactic acid bacteria (LAB) are predominantly utilized [16,17]. LAB, integral to the human gut microbiome [18], have been implicated in various health benefits, including for the prevention and treatment of cancer [19], diabetes [20], and obesity [21] and as anti-allergens [22] and anti-inflammatories [23]. Notably, LAB have demonstrated positive effects on IBD improvement. Zhang et al. [24] demonstrated that *Lactobacillus gasseri* G098 mitigates DSS-induced colitis in mice by diminishing mucosal injury, adjusting immune reactions, reinstating gut microbiota diversity, and promoting microbiota stability. A study by Hevia et al. [25] explored the levels of antibodies (IgG and IgA) raised against extracellular proteins produced by LAB in association with IBD, while Scaldaferri et al. [26] explored Lactobacillus GG’s efficacy as an adjuvant in maintaining remission in CD patients. Despite the extensive attention garnered by the potential therapeutic effects of probiotics in treating IBD, significant research gaps persist in clinical practice, leaving ample room for exploring probiotic strains, therapeutic mechanisms across different diseases, and related aspects.

This review delves into the intricate interplay between symptomatic features of IBD, influencing factors, and their connection to gut microbes, with a specific emphasis on the pivotal role of LAB in preventing and treating IBD. We highlight distinctions in the treatment of UC and CD using LAB, offering novel insights for future IBD treatment strategies.

## 2. Pathogenesis

**Figure 2:**
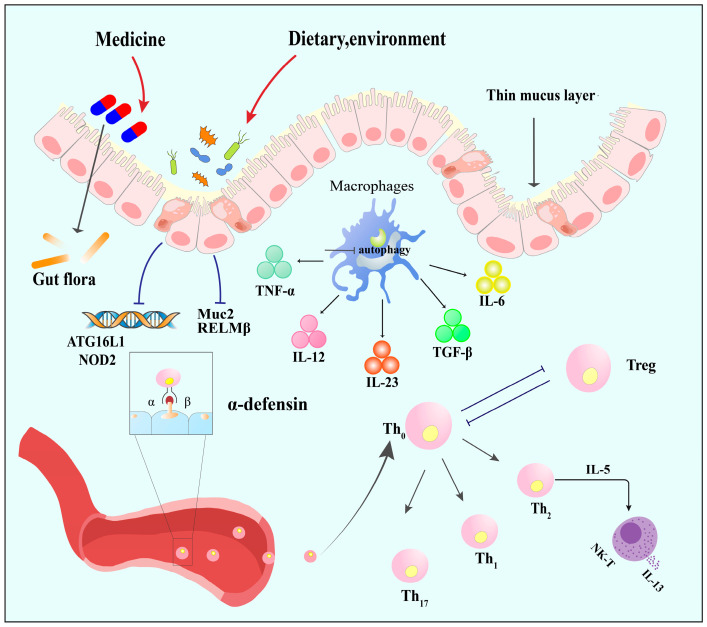
Inflammatory bowel disease pathogenesis. Risk factors trigger microbial dysbiosis in IBD, reducing SCFA-producing bacteria and increasing Proteobacteria. Intestinal barrier integrity is compromised by decreased E-cadherin, altered goblet cell function (Muc2, RELMβ), and Paneth cell dysfunction (NOD2, ATG16L1). Innate immune dysregulation involves reduced CD14+ macrophages and impaired autophagy. An imbalance between effector and Treg leads to uncontrolled T cell activation and abnormal leukocyte migration in the inflamed intestine. Abbreviations: NOD2: nucleotide-binding oligomerization domain 2; ATG16L1: autophagy related 16 like; Muc2: mucin 2; RELMβ: resistin-like molecule β; TNF-α: tumor necrosis factor-α; IL: interleukin; TGF-β: transforming growth factor-β; Th: helper T cell; Treg: regulatory T cell; NK-T: natural killer/T cell.

IBD represents a chronic inflammatory disorder affecting the gastrointestinal tract, which clinically contains CD, UC, and other related disorders [7]. While the precise etiology of IBD remains incompletely elucidated, multiple contributory factors are thought to impact disease activity. These factors include host genetic predispositions, geographic considerations, and dietary influences (Figure 2).

### 2.1. Genetic Factors

Genome-wide association and next-generation sequencing studies have identified over 240 distinct genetic risk loci, with approximately 30 loci common to both CD and UC [27,28,29]. Among these loci, NOD2, located on chromosome 16, exhibits the strongest association with IBD and is particularly associated with CD [30,31]. I NOD2 is a cytosolic protein expressed in various leukocytes, gut epithelial cells (including Paneth cells), and lamina propria lymphocytes, such as T cells [32,33,34,35]. Upon ligand binding, NOD2 oligomerizes, initiating the activation of nuclear factor κB (NF-κB) and mitogen-activated protein kinase pathways, which in turn induce the transcription of inflammatory cytokines [36,37].

Other than genetic risk loci, Recent research has also highlighted DNA methylation and noncoding RNAs in the onset and course of IBD [38,39]. Current findings suggest that epigenetic modifications are present in IBD patients, as evidenced by the differential expression of specific microRNAs (miRNAs) in colonic mucosa samples from IBD patients compared to controls [40,41]. Dysregulation of miRNAs in Th17 cells has also been linked to IBD pathogenesis. Notably, specific miRNAs such as miR-16, miR-21, and miR-223 show increased expression in active IBD cases compared to those in remission, despite the overall miRNA levels remaining constant [38,42]. The exploration of genes (NOD2, ATG16L1, LRRK2, etc.) associated with bacterial sensing, innate immunity, and Th17 cell function, along with alterations in mucus layers, has significantly linked these genetic factors to the development of CD [43,44]. In addition, defects in autophagy-related genes such as ATG16L1 and IRGM have also been identified as crucial risk factors for CD [43]. These findings underscore the intricate interplay of genetic and epigenetic factors in shaping the landscape of IBD susceptibility and progression.

### 2.2. Environmental Factors

The escalating prevalence of IBD attributed to industrialization, coupled with an augmented risk observed among individuals relocating to regions with higher IBD prevalence, underscores the significance of environmental factors in IBD development [45]. Ananthakrishnan et al. [46] examined the correlation between air pollution and IBD incidence in Wisconsin, USA. They found a 40% increase in IBD hospitalizations per 1 log increase in total criteria pollutant emissions, with carbon monoxide, nitrogen dioxide, sulfur dioxide, and PM2.5, significantly associated with IBD. In addition, living near heavy traffic was linked to a higher risk of IBD, and other pollutants like nitrous oxides showed a tendency toward positive correlations with the disease [47].

Beyond air pollution, the impact of cigarette smoking on IBD susceptibility is noteworthy. Smoking has been reported to significantly elevate the susceptibility to and exacerbate CD, but in contrast, it has a protective effect on the development of UC and reduces its severity [48]. On the one hand, smoking affects thiopurine metabolism and thus influences the time to disease recurrence [49], and on the other, research has shown that smoking worsens inflammation by modifying the relative abundance of certain gut microbes. Specifically, there is an increase in *Ruminococcus gnavus* and *Bacteroides vulgatus* and a decrease in *Faecalibacterium prausnitzii* and *Akkermansia muciniphila* [50]. Furthermore, a randomized trial involving 17 patients who underwent bowel resection and received long-term treatment revealed that smoking increases the risk of postoperative recurrence in patients with CD, necessitating surgical intervention [51]. In summary, these findings underscore the multifaceted role of environmental factors, such as air quality and smoking habits, in modulating IBD risk and outcomes.

### 2.3. Medication

While host genetics may partially influence the structure of gut microbiota, medication from birth through adulthood persistently modifies the composition, organization, and function of the gut microbiome, thus continuously affecting the disease risk throughout an individual’s lifespan [52,53]. Antibiotics, commonly used against pathogenic organisms during disease, lack the ability to discriminate between normal flora and pathogenic bacteria, which may lead to the depletion of gut microbiota and consequent health disorders [54]. Bacteria in the colon, particularly anaerobes, play a crucial role in metabolizing undigested carbohydrates into lactic acid and short-chain fatty acids (SCFAs) [55].

Butyric acid serves as a vital energy source for the mucosal layer; its deficiency compromises the integrity of the colonic epithelium, impairs mucosal function, and affects T cell regulation, thereby playing a significant role in the onset of IBD [56,57,58]. Petra Zimmermann and colleagues conducted a study revealing that antibiotics significantly impact the composition of gut microbiota [59]. Notably, antibiotics commonly used for IBD, such as amoxicillin, cephalosporins, macrolides, clindamycin, and quinolones were found to diminish the count of *Escherichia coli* [60,61,62,63,64,65,66], and treatment with amoxicillin [67], paromycin [68], clindamycin [69], and minocycline [69] reduced the number of *E. faecalis* spp. These findings highlight the influence of medication, particularly antibiotics consumption, on the delicate balance of the gut microbiota, underscoring their potential implications for IBD development.

### 2.4. Dietary Factors

Among the factors associated with IBD, diet is easily modifiable, presenting itself as a promising target for both prevention and treatment. Multiple studies have shown that a higher intake of fiber, fruits, and vegetables is linked to a reduced risk of IBD [70]. Conversely, diets that are high in meat and fats, especially those containing polyunsaturated fatty acids and omega-6 fatty acids, are associated with an increased risk [71]. A study by Julia Fritsch et al. demonstrated that a low-fat, high-fiber diet (LFD) resulted in reduced inflammatory markers and alleviated gut microbial dysregulation, indicating potential benefits for individuals with UC in remission [72]. Other investigations by Marton et al. [73] and Eleonora Scaioli et al. [74] demonstrated a notable elevation in omega-6 (ω6) fatty acids alongside a reduction in omega-3 (ω3) fatty acids within the colonic mucosa of patients suffering from active UC. Moreover, findings from a large-scale prospective multicenter cohort study that included over 200,000 participants indicated that an increased dietary intake of ω3 fatty acids was linked to a diminished risk of developing UC [75]. In addition, diet can influence the course of IBD by altering gut microbes. Resveratrol, a polyphenolic compound found in the diet, has been observed to mitigate dysbiosis in the gut microbiota caused by colitis. This microbiota plays a protective role against colon inflammation by promoting the induction of regulatory Treg cells and inhibiting the activity of pro-inflammatory Th1/Th17 cells [76].

The results of this study highlight the essential influence of dietary habits in the pathogenesis of IBD. Diet plays a significant role in modulating the immune system, inflammatory reactions, and gut microbiota. Furthermore, dietary components directly interact with gut mucosal defenses and inflammatory cells [77,78]. Comparisons between healthy populations and IBD patients reveal significantly lower bacterial diversity (e.g., bacillus-like organisms) both in inflamed intestinal segments and segments without inflammation [79]. Thus, probiotics, particularly LAB, have gained attention for their potential to modulate the gut microbiota and immune system, offering new perspectives for IBD treatment.

## 3. The Role of LAB in Inflammatory Bowel Disease

Under physiological conditions, the gut microbiota sustains a mutualistic and symbiotic relationship with the host. Alongside the intestinal epithelial barrier and immune cells, it constructs a complex gut microbial ecosystem. This ecosystem not only aids in systemic metabolism but also plays a crucial role in maintaining gut homeostasis [80]. The gut microbiota engages dynamically with host physiological processes, aiding in the metabolism of carbohydrates and amino acids [81], protection against epithelial cell damage [82], energy harvest and storage from the diet [83], and induction of intestinal angiogenesis [84]. Probiotics may confer health benefits by enhancing the synthesis of SCFAs and lactate. These compounds suppress the proliferation of potentially harmful microorganisms and exhibit anti-inflammatory properties in the gastrointestinal tract [85]. This infers that LAB could play an active role in the prevention and treatment of IBD, offering novel perspectives for therapeutic interventions. It should be emphasized that IBD includes both UC and CD, each displaying unique traits. The effectiveness of LAB in treating IBD may not be uniform across both conditions.

### 3.1. The Role of LAB in UC

UC represents one of the two primary forms of IBD and is a chronic inflammatory condition affecting the colonic mucosa. The disease begins in the rectum and progresses proximally through the colon, marked by recurrent episodes of flare-ups and periods of remission [4,86]. The pathogenesis of UC is closely linked to defects in colonic epithelial cells (colonocytes), the mucus barrier, and the epithelial barrier. In recent years, an increasing number of LAB strains are being employed in the treatment of UC, with their therapeutic rationale encompassing the following key aspects.

#### 3.1.1. Improvement of Intestinal Barrier Function

The intestinal mucosal barrier is composed of various intricate elements, primarily including a mucus layer on the surface, an epithelial cell stratum, and a basement membrane of the mucosa. It functions as the first line of defense against external threats, significantly contributing to the reduction of pathogen invasion and toxin absorption. Johansson et al. [87] observed a decrease in the number of goblet cells and a reduction in the mucus layer thickness in patients suffering from active UC. This condition was also characterized by diminished mucus adhesion to the intestinal wall, facilitating bacterial infiltration through the mucus and enabling them to reach the epithelial cells.

Belo GA. et al. [88] examined the alleviative effects of the surface-layer protein B from *Propionibacterium freudenreichii* CIRM-BIA 129 in conjunction with Lactobacillus lactis NCDO 2118 on DSS-induced colitis in mice. Their study reported an increase in muc-2 gene expression, a restoration of goblet cells producing protective mucus on the intestinal mucosa, and an enhancement in the expression of tight junction proteins in the colon. These changes collectively resulted in elevated levels of anti-inflammatory cytokines and reduced levels of pro-inflammatory cytokines in the colons of colitis-afflicted mice. Clover C M Wong et al. found that mCRAMP-encoded *Lactobacillus lactis* restored the mucus-secreting layer and also promoted the crypt integrity, which was driven by persistent inflammation in IBD [89,90]. These studies collectively suggest that LAB contribute to mucosal repair in IBD patients. However, specific therapeutic effects require further research and clinical validation for a comprehensive understanding of their efficacy.

#### 3.1.2. Inhibition of Inflammatory Response

**Figure 3:**
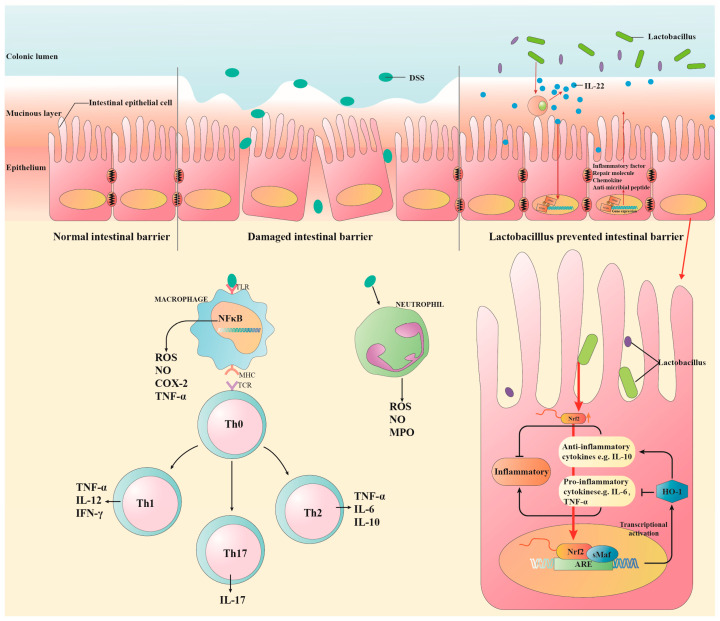
The role of LAB in UC. LAB treats and ameliorates UC primarily by improving the intestinal barrier, inhibiting the expression of cytokines, and modulating the gut microbiota. Abbreviations: ROS: reactive oxygen species; NO: nitric oxide; COX-2: cyclooxygenase-2; NF-κB: nuclear factor κ-light-chain-enhancer of activated B cells; IFN-γ: interferon-γ; MPO: myeloperoxidase; Nrf2: nuclear factor erythroid 2-related factor 2; HO-1: heme oxygenase-1; sMaf: small Maf transcription factor; ARE: antioxidant response element.

UC is also characterized by an uncontrolled immune response and an imbalance in cytokine release, leading to dysregulation of pro-inflammatory cytokines (e.g., TNF-α, IL-6, IL-1β, and IFN-γ) and anti-inflammatory cytokines (e.g., IL-10) [91]. Therefore, achieving equilibrium among these inflammatory mediators is regarded as a potential therapeutic approach for managing UC. LAB were reported to modulate the immune system’s response, produce anti-inflammatory cytokines that inhibit the inflammatory response and alleviate UC symptoms [92,93] (Figure 3).

Hao Haining et al. [92] found that *Lactobacillus plantarum* Q7-derived extracellular vesicles down-regulated the expression of TLR4 and MyD88 genes in DSS-induced colitis mice, leading to the phosphorylation of NF-κB, ameliorating colitis by regulating inflammatory cytokines such as IL-1β, IL-6, and TNF-α. Similarly, El-Baz et al. [93] fed rats with a suspension containing LAB and reported that both Nrf2 and HO-1, which modulate inflammation and promote angiogenesis in UC, were significantly increased compared to the standard therapy 5-aminosalicylic acid.

Another study reported by Dong et al. [94] demonstrated that administration of *Pediococcus pentosaceus* CECT 8330 mitigated DSS-induced colitis in mice. This protection was evidenced by a reduction in serum proinflammatory cytokines (TNF-α, IL-1β, and IL-6) and an elevation in IL-10 levels. Additionally, the treatment elevated fecal SCFAs and boosted the relative abundance of beneficial bacterial genera such as *Lactobacillus* and *Bifidobacterium*. Wang et al. [95] identified that a combination of *Lactobacillus* strains could serve as a promising therapeutic approach to prevent carcinogenesis associated with UC by influencing gut microbiota and reducing the concentrations of TNF-α and IL-6 in colon tissue.

#### 3.1.3. Regulation of Gut Microorganisms

Recent research has highlighted the crucial impact of gut microbiota dysbiosis on the advancement of UC. This condition is characterized by a notable reduction in microbial diversity and a diminished presence of beneficial bacteria, such as those belonging to the phylum Bacteroides. Concurrently, there is an observed increase in pathogenic bacteria, including *Escherichia coli* and specific anaerobic species [96,97]. In a study by Liu Jialing et al. [98], differences in the composition and abundances of gut microbiota were observed in a DSS-induced UC model, with significant decreases in *Lactobacillus* and *Alistipes* and significant increases in *Oscillibacter*, *Streptococcus*, and *Escherichia Shigella*, while LAB has demonstrated the ability to restore a healthy gut microbial ecosystem by enriching beneficial bacteria in the intestine, altering the microbial structure, reducing pathogenic bacteria, and strengthening the intestinal mucosal barrier function [99,100,101,102]. A recent study by Olekhnovich et al. [103] reported that the administration of *Levilactobacillus brevis* 47f to rats increased the abundance of SCFA-producing bacteria such as *Faecalibacterium* and *Roseburia*. Furthermore, research conducted by Min Deng et al. [104] revealed that *Lactobacillus paracasei* L9 enhances the population of butyrate-producing bacteria, including Lachnospiraceae and Ruminococcaceae. These bacteria suppress the IL-6/STAT3 signaling pathway, thereby reducing the inflammatory response in colitis induced by DSS. Similarly, You Jin Jang et al. [105] administered *Lactobacillus fermentum* KBL374 and KBL375 to DSS-treated mice. This intervention resulted in a higher abundance of *Lactobacillus* and *Akkermansia* species, which contributed to enhanced IL-10 production and improved mucus layer integrity, ultimately mitigating DSS-induced colitis. All of the above studies confirm the significant role of LAB in the treatment and prevention of UC. It is essential to recognize potential variations in efficacy based on individual differences. LAB treatment mainly focuses on regulating gut microbiota and reducing inflammatory response, with potential limitations in addressing other UC symptoms, such as abdominal pain and diarrhea.

### 3.2. The Role of LAB in CD

CD, a persistent infectious condition affecting the gastrointestinal system, has seen a global rise in incidence in recent years. Much like UC, the development of CD is attributed to the interplay between gut microbiota, environmental influences, and modified genetic predisposition, culminating in the disruption of the body’s innate and adaptive immune responses. Despite sharing a chronic inflammatory state and some symptoms with UC, there are notable differences between the two diseases.

Gut microbiota dysbiosis CD in patients is mainly characterized by a decrease of bacillus-like and thick-walled bacteria, particularly Clostridium groups XIVa and IV, and an increase in gamma-proteobacteria and actinomycetes [106]. A significant proportion, roughly one-third, of CD patients demonstrate an elevated presence of adherent-invasive *Escherichia coli*. This bacterium penetrates the mucosal barrier, infiltrates intestinal epithelial cells, persists within macrophages, and stimulates increased secretion of TNF-α. This cascade of events results in inflammation and subsequent damage to the mucosal barrier [107,108]. Several studies reported that probiotic strains could modulate the release of intestinal epithelial cytokines and inhibit the production of the transcription factor NF-κB in immune system cells, leading to a reduction in intestinal inflammation [109,110]. Sara Notararigo et al. [111] found that *Lactobacillus* and *Pediococcus* strains isolated from alcoholic beverages were shown to synthesize O2-substituted-(1-3)-β-D-glucan, which could protect the intestine by regulating NF-κB in an in vitro model of CD biopsy. Additionally, in a mouse experiment by Jean Guy LeBlanc et al. [112], *Lactobacillus casei* BL23, producing CAT and SOD, reduced trinitrobenzene sulfonic acid (TNBS)-induced damage in mice with CD, with a significant increase in survival, decreased bacterial translocation to the liver, and prevention of colonic injury. These results underscore the potential role of LAB in mitigating inflammation and mucosal injury linked to CD, thereby offering a promising therapeutic approach. Although most evidence comes from animal model studies, the clinical application of LAB in IBD treatment has been gradually expanding. Table 1 details the use of lactobacillus in clinical trials. However, their efficacy varies between UC and CD, highlighting the need for further research to better understand their therapeutic impact.

In summary, LAB shows substantial therapeutic promise in both UC and CD by enhancing intestinal barrier integrity, modulating immune responses, and rebalancing gut microbiota. These findings highlight the potential of LAB in reducing chronic inflammation and mucosal damage in IBD.

### 3.3. Distinctions in the Application of LAB for UC and CD

Numerous clinical trials have firmly confirmed the effectiveness of probiotics in preventing pouchitis and sustaining remission in ulcerative colitis UC. However, the evidence supporting the role of probiotics in CD remains inadequate, lacking clear criteria for strain selection, dosage, and regimen prescription. Few studies have investigated the impact of probiotics on the induction and maintenance of remission in CD, with a significant lack of extensive randomized controlled trials. Emerging data indicate that LAB contribute to the prevention of UC, including strains such as VSL#3 [119], *Lactobacillus* [120], and *Bifidobacterium* [121]. Nevertheless, the evidence supporting LAB for therapeutic and preventive purposes in CD is inconclusive. Systematic reviews and meta-analyses [122,123,124] consistently suggest that probiotics, including *Saccharomyces boulardii*, *Lactobacillus*, and *Bifidobacterium*, do not exhibit significant efficacy in inducing or maintaining remission in CD patients.

**Figure 4:**
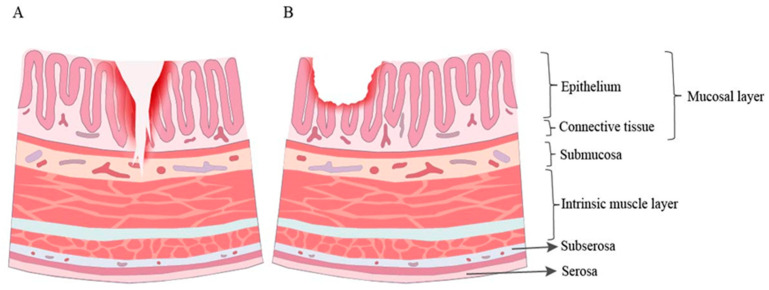
Differences between UC and CD pathogenesis. In UC (**A**), lesions are primarily confined to the mucosal layer, characterized by superficial ulcers and crypt abscesses. Conversely, CD (**B**) is marked by segmental inflammation with transmural involvement, presenting as deep fissuring ulcers and granulomatous inflammation.

The disparity in efficacy of LAB treatment between UC and CD may stem from various factors. In terms of disease onset, UC typically manifests in a localized manner in the intestine, primarily in the sigmoid colon and rectum. While CD can exhibit a segmented presence along the whole gastrointestinal tract, it frequently impacts the terminal ileum and colon [2] (Figure 4). The heightened innate immune pathways observed in UC, particularly TLR4- and TLR2-mediated pathways, suggest that probiotic therapies could potentially restore specific bacterial species inducing regulatory T cells in the gut, as UC exhibits a more active pattern recognition-mediated antibacterial immunity [125]. This suggests that microbiome-related immunity may play a more prominent role in UC, potentially influencing the efficacy of probiotic therapies.

In summary, the inconsistency in probiotic studies for IBD, especially CD, under-scores the need for further exploration. Research avenues should include: (1) individualized treatment: tailoring probiotic strains and doses for different IBD patient profiles; (2) combination therapy: exploring combinations of multiple probiotic strains to enhance survival and therapeutic effects; (3) fecal microbiota transplantation: investigating the transfer of high-quality healthy gut microbiota to IBD patients for symptom improvement; (4) combined therapies: exploring synergies between probiotics and other treatments (e.g., antibiotics, immunosuppressants, glucocorticoids) for enhanced outcomes. As medical technology advances and a deeper understanding of IBD treatment mechanisms emerges, the future outlook for probiotics in IBD treatment appears promising.

## Figures and Tables

**Table 1:** Clinical Trials of LAB in IBD.

Disease	Probiotic Types and Dosage	Benefits	Reference
UC	9 Bio-Three tablets (2 mg *Streptococcus faecalis* T-110, 10 mg *Clostridium butyricum* TO-A, 10 mg *Bacillus enterocolitica* TO-A) daily for 12 months	Probiotics effective in maintaining clinical remission in resting UC patients	[113]
	A single daily oral dose of mesalazine 1200 mg and a probiotic mixture (consisting of *Lactobacillus salivarius*, *Lactobacillus acidophilus*, and *Bifidobacterium bifidum* BGN4) twice daily for 2 years	The combination therapy group showed better improvement compared to the control group (1200 mg of oral mesalazine daily), and the beneficial effects of probiotics were evident even after two years of treatment	[114]
	After 48 h of incubation under aerobic conditions, *Lactobacillus kefiri* CIDCA 8348 was resuspended in PBS at a concentration of approximately 1–2 × 10^8^ CFU/mL	*L. kefiri* reduced the secretion of TNF-α, IL-6, IFN-γ, and IL-13 in patients. It also induced an increase in the frequency of CD4FOXP3 lamina propria T cells and an increase in IL-10 levels	[115]
	100 mL of *Lactobacillus helveticus* CP790-fermented milk (1 × 10^10^ CFU/100 mL) per day for 4 weeks	Ingestion of *Lactobacillus helveticus* CP790 reduced the abundance of Desulfovibrio vulnificus and improved constipation symptoms in subjects, as well as improving overall mood and depression in healthy individuals	[116]
CD	VSL#3 sachets (one sachet containing 450 billion live bacteria twice daily) for 90 days, followed by open-label VSL#3 (one sachet twice daily) for up to 12 months	While there was no statistical difference in endoscopic recurrence rates at day 90 between VSL#3 and placebo, lower mucosal cytokine levels and reduced recurrence rates were observed with long-term VSL#3 use	[117]
	Daily oral mesalazine enteric-coated tablets (1 g/day, 3 times/day) and probiotic combination live capsules (*Bacteroides vulgatus* ≥ 1.0 × 10^6^ CFU, *Lactobacillus acidophilus* ≥ 1.0 × 10^6^ CFU, and *Enterococcus faecalis* ≥ 1.0 × 10^6^ CFU) for 4 weeks	Compared with the control group, the observation group had higher numbers of *Lactobacillus acidophilus* and *Bifidobacterium Longum*, higher levels of serum IL-10, higher levels of peripheral blood CD4+ and CD4+/CD8+, and a higher total clinical effectiveness rate than the control group	[118]

## Data Availability

No new data were created or analyzed in this study.
